# Chicken ANP32A-independent replication of highly pathogenic avian influenza viruses potentially leads to mammalian adaptation-related amino acid substitutions in viral PB2 and PA proteins

**DOI:** 10.1128/jvi.01840-24

**Published:** 2024-11-21

**Authors:** Yoshikazu Fujimoto, Kinuyo Ozaki, Etsuro Ono

**Affiliations:** 1 Transboundary Animal Diseases Research Center, Joint Faculty of Veterinary Medicine, Kagoshima University 12851 , Kagoshima, Japan; 2 Joint Graduate School of Veterinary Medicine, Kagoshima University 12851 , Kagoshima, Japan; 3 Center of Biomedical Research, Research Center for Human Disease Modeling, Graduate School of Medical Sciences, Kyushu University 12923 , Fukuoka, Japan; 4 Department of Biomedicine, Graduate School of Medical Sciences, Kyushu University 12923 , Fukuoka, Japan; St. Jude Children's Research Hospital, Memphis, Tennessee, USA

**Keywords:** avian viruses, veterinary pathogens, viral replication

## Abstract

Acidic nuclear phosphoprotein 32 family member A (ANP32A) is an important host factor that supports the efficient replication of avian influenza viruses (AIVs). To develop an antiviral strategy against Gs/Gd-lineage H5 highly pathogenic avian influenza (HPAI) viruses in chickens, we established chicken ANP32-knockout (chANP32A-KO) DF-1 cells and evaluated their antiviral efficacy through *in vitro* validation. The replication of all HPAI viruses tested in chANP32A-KO cells was significantly lower compared to that of wild-type DF-1 cells. However, when HPAI strains A/mountain hawk-eagle/Kumamoto/1/2007 (H5N1; MHE) and A/chicken/Aichi/2/2011 (H5N1; H5Aichi) were passed in chANP32A-KO cells, mutant viruses were generated, which exhibited comparable replication levels in both chANP32A-KO and wild-type DF-1 cells. Sequence analysis revealed that mammalian-adaptive amino acid mutations PB2_D256G and PA_T97I were present in the MHE mutant virus, and the PB2_E627K mutation was identified in the H5Aichi mutant virus. These mutations have also been reported to enhance the polymerase activity of AIVs in mammalian cells; however, the minigenome assay in the present study showed that the polymerase activity of mutant viruses in chANP32A-KO cells was not restored to levels comparable to those in wild-type DF-1 cells. These findings suggest that ANP32A-independent viral replication may induce amino acid substitutions associated with mammalian adaptation in AIVs. They also imply that the high efficiency of viral replication mediated by these amino acid mutations may not result from enhanced polymerase activity but rather involve other undefined mechanisms.

**IMPORTANCE:**

During the host-switching of avian influenza viruses (AIVs) to mammalian hosts, introducing adaptive mutations into viral proteins is essential to ensure optimal functionality through virus-host protein interactions in mammalian cells. However, the mechanisms leading to adaptive mutations in viral proteins remain unclear. Among several host proteins that promote viral growth, acidic nuclear phosphoprotein 32 family member A (ANP32A) is known to be an important factor for efficient viral replication. Here, we generated mutant highly pathogenic avian influenza viruses capable of ANP32A-independent replication in a chicken-derived cell line. We demonstrated that several amino acid mutations found in the mutant viruses correspond to those associated with the mammalian adaptation of AIVs. These results suggest that ANP32A-independent viral replication is one of the mechanisms for introducing amino acid mutations that are reportedly involved in the mammalian adaptation of AIVs.

## INTRODUCTION

A/Goose/Guangdong/1996 (Gs/Gd)-lineage H5 highly pathogenic avian influenza (HPAI) viruses have circulated among migratory wild birds for over 25 years. The threat of HPAI outbreaks poses a significant concern for the global poultry industry. Vaccination strategies have been extensively employed in Gs/Gd-lineage H5 HPAI-enzootic countries to mitigate economic losses in poultry production, but they have proven insufficient (1). One method to control HPAI in poultry is via genetic modification to introduce novel genes that interfere with viral infection or to delete genes associated with viral replication (2, 3). Long et al. (4) demonstrated that acidic nuclear phosphoprotein 32 family member A (ANP32A) is an important host factor that supports the viral polymerase activity of influenza A virus isolates from a wide range of hosts. This finding suggests that ANP32A may become a candidate host target for novel antivirals. With the long-term goal of producing HPAI-resistant poultry, we examined the antiviral effects of chicken ANP32A (chANP32A) deletion against Gs/Gd-lineage HPAI virus infections in chicken-derived cells. During the course of experiments, mutant viruses capable of replicating effectively in chANP32A-knockout (KO) cells were unexpectedly selected. Sequence analysis revealed that some substitutions observed in PB2 and PA genes of these mutant viruses were identical to mutations found in mammalian-adapted viruses. These findings are important for assessing the risk of evolutionary HPAI virus emergence before the practical application of genetically engineered chickens. Here, we discuss the potential of chANP32A gene-edited chickens for avian influenza control.

## RESULTS AND DISCUSSION

### Replication of Gs/Gd-lineage HPAI viruses is suppressed in a chANP32A-KO DF-1 cell line

**Fig 1:**
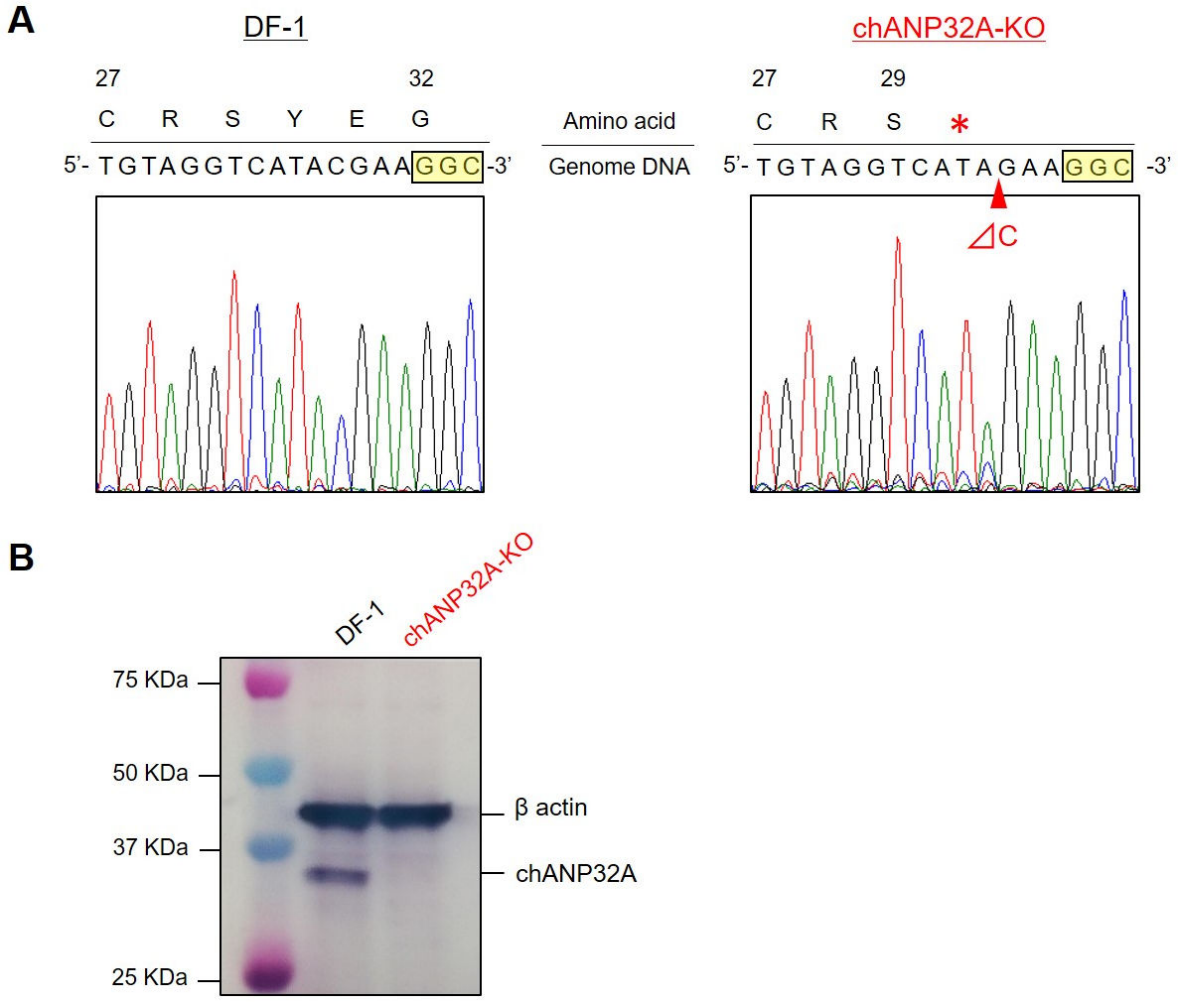
Generation of chANP32A-KO DF-1 cells. (**A**) Nucleotide sequence analysis of a gene-editing target site. Comparison between wild-type DF-1 and chANP32A-KO cells showed that a cytosine base located four bases upstream of the 5′-side of the PAM sequence (yellow box) was deleted in exon 2 of chANP32A in the genomic DNA of chANP32A-KO cells, resulting in the insertion of a termination codon (*) immediately after amino acid position 29 of chANP32A. (**B**) Detection of the chANP32A protein in cell lysates from wild-type DF-1 and chANP32A-KO cells by Western blotting using anti-ANP32A-specific polyclonal antibodies. A band with a molecular weight of approximately 32 KDa, corresponding to the size of chANP32A, was observed in the lysate sample from wild-type DF-1 but not in chANP32A-KO cells.

The avian ANP32 family comprises ANP32A, ANP32B, and ANP32E; however, only ANP32A can effectively support the viral polymerase activity and replication of avian influenza viruses (5, 6). To establish chANP32A-KO chicken-derived cells, a genetically modified DF-1 clone was generated using CRISPR/Cas9 genome editing. CRISPR target sequences were selected from exon 2 of the chANP32A gene by use of CRISPRdirect (https://crispr.dbcls.jp). Sequence analysis of genomic DNA from the genetically modified cell clone revealed a one-base deletion 4 bp upstream of the protospacer adjacent motif (PAM) sequence. This deletion resulted in a frameshift mutation and the introduction of a stop codon immediately after the amino acid at position 29 (Fig. 1A). Western blot analysis revealed that endogenous chANP32A was not expressed in chANP32A-KO cells (Fig. 1B).

**Fig 2:**
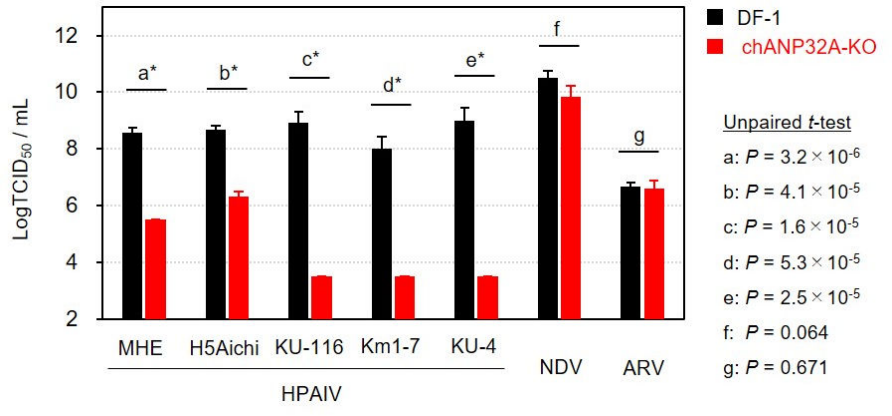
Resistance to HPAI virus infections in chANP32A-KO cells. Cells were seeded in 48-well plates, inoculated with HPAI viruses, and observed for cytopathic effects at 3 days post-inoculation. Viral titers were calculated using the Reed and Muench method and expressed as the mean of three independent replicates. Each bar is marked with the corresponding standard deviation. Statistical analysis of the differences in viral titers between wild-type DF-1 and chANP32A-KO cells was performed using an unpaired *t*-test. Asterisks indicate statistically significant differences (*P* < 0.05). NDV, Newcastle disease virus; ARV, avian reovirus.

To determine whether the chANP32A deletion confers resistance to HPAI virus infection, we compared viral replication between wild-type DF-1 cells and chANP32A-KO cells using a 50% tissue culture infectious dose (TCID_50_) assay. Five Gs/Gd-lineage H5 HPAI virus strains were tested: MHE (H5N1), H5Aichi (H5N1), KU-116 (H5N8), Km1-7 (H5N8), and KU-4 (H5N6). For each strain, the mean virus titer was significantly reduced in chANP32A-KO cells compared to wild-type DF-1 cells (Fig. 2). The mean virus titers of all tested viruses ranged from 8.0 to 9.0 log TCID_50_/mL^−1^ in DF-1 cells. In chANP32A-KO cells, the mean titers of MHE and H5Aichi were 5.5 and 6.3 log TCID_50_/mL^−1^, respectively, while those of KU-116, Km1-7, and KU-4 strains were 3.5 log TCID_50_/mL^−1^. Virus titers were also measured for two poultry pathogens, Newcastle disease virus and avian orthoreovirus; neither showed any significant difference in titer between chANP32A-KO cells and wild-type DF-1 cells (Fig. 2), suggesting that the chANP32A deletion specifically affects avian influenza virus replication in chicken-derived cells.

### Repeated passaging of the Gs/Gd-lineage HPAI virus in chANP32A-KO cells generates mutant viruses capable of ANP32A-independent replication

**Fig 3:**
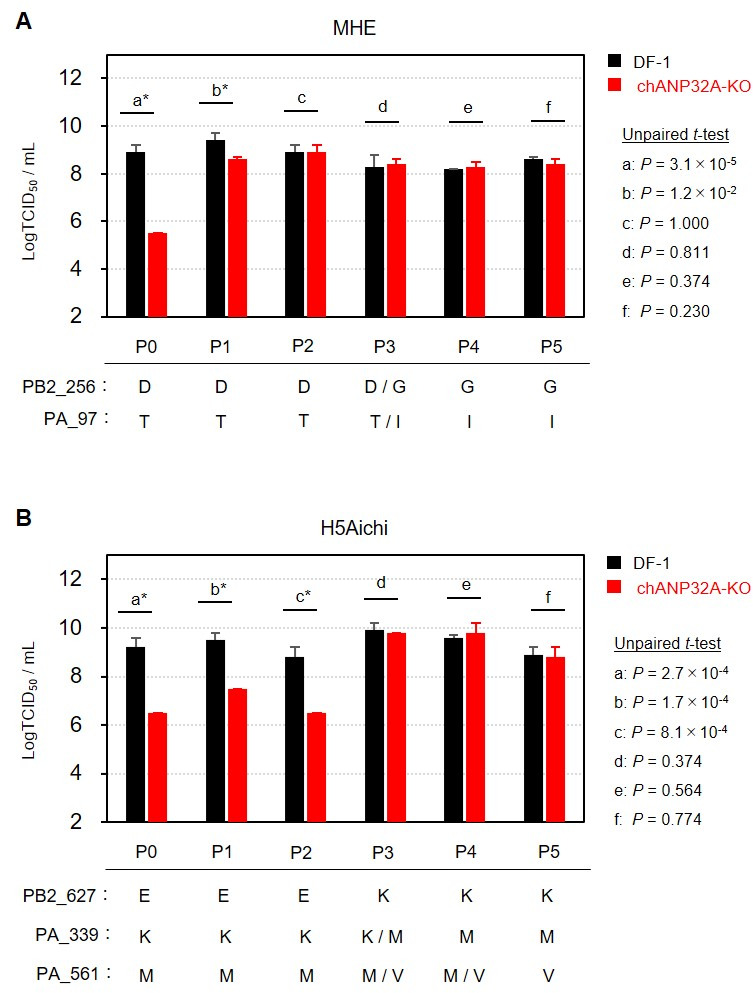
Virus titers of mutant HPAI virus in wild-type DF-1 and ANP32A-KO cells. Comparison of the viral titers of the mutant MHE (**A**) and H5Aichi strains (**B**) in wild-type DF-1 and chANP32A-KO cells. These mutant viruses were generated via serial passage (P1 to P5) in chANP32A-KO cells. After each virus was propagated once in embryonated chicken eggs, the virus titer was determined by a TCID_50_ assay. Sequence analysis revealed amino acid mutations at positions 256 of the PB2 and 97 of the PA proteins in the MHE-mutant strains (**A**) and positions 627 of the PB2 and 339 and 561 of the PA proteins in the H5Aichi-mutant strains (**B**). Statistical analysis of the differences in viral titers between wild-type DF-1 and chANP32A-KO cells was performed using an unpaired *t*-test. Asterisks denote statistically significant differences (*P* < 0.05).

Another research group reported that viral replication of a field-isolated Gs/Gd-lineage virus was reduced in ANP32A-KO avian cell lines compared to wild-type cells; however, viral growth was not completely abolished in ANP32A-KO cells (6). Consistent with these findings, in this study, the viral titers of all tested Gs/Gd-lineage HPAI viruses in chANP32A-KO cells were at least 3.5 log TCID_50_ mL^−1^ (Fig. 2). These results indicate that certain avian influenza viruses are capable of chANP32A-independent replication although the replication efficiency varies between strains. To further investigate chANP32A-independent viral replication and to elucidate any adaptations related to escaping the adverse effects of chANP32A deletion, we serially passaged each tested HPAI virus strain in chANP32A-KO cells. This experiment is important for determining whether virus mutations could potentially occur in infected chANP32-KO chickens in the field. The KU-116, Km1-7, and KU-4 strains proved to be unrecoverable from the inoculated chANP32A-KO cell supernatants by the end of the second passage (data not shown); however, the MHE and H5Aichi strains were successfully recovered from chANP32A-KO cells through five serial passages and were subsequently subjected to further evaluation and sequencing. The titers of the MHE strain significantly differed between chANP32A-KO and wild-type DF-1 cells in the parental strain (unpassaged, P0) and at Passage 1 (P1); however, the magnitude of this difference decreased from 3.4 to 0.8 log TCID_50_ mL^−1^ (Fig. 3A). From Passage 2 to Passage 5 (P2 to P5), there was no significant difference in titers between chANP32A-KO and wild-type DF-1 cells (Fig. 3A). The titers of the H5Aichi strain significantly differed between chANP32A-KO and wild-type DF-1 cells at P0, P1, and P2, with differences ranging from 2.0 to 2.7 log TCID_50_ mL^−1^. However, no such differences were observed from P3 to P5 (Fig. 3B). These results suggest that, after several passages, the mutant strains of MHE and H5Aichi exhibited similar replicative potential in avian-derived cells, regardless of chANP32A deletion.

Whole-genome sequencing revealed mutations in both passaged strains. In the MHE strain, we identified a substitution of aspartic acid with glycine at residue 256 in the PB2 subunit, as well as a substitution of threonine with isoleucine at residue 97 in the PA subunit (P3 to P5: PB2_D256G and PA_T97I, respectively) (Fig. 3A). In the H5Aichi strain, we identified a substitution of glutamic acid with lysine at residue 627 in the PB2 subunit, a substitution of lysine with methionine at residue 339, and a substitution of methionine with valine at residue 561 in the PA subunit (P3 to P5: PB2_E627K, PA_K339M, and PA_M561V, respectively) (Fig. 3B). Among the five mutations identified (PB2_D256G and PA_T97I in MHE-P5, and PB2_E627K, PA_K339M, and PA_M561V in H5Aichi-P5), three have previously been associated with mammalian adaptations. The PB2_D256G mutation has been identified in human- and swine-adapted avian influenza viruses, while PA_T97I has been observed in mouse-adapted avian influenza viruses (7–11). PB2_E627K is the most common mutation found in mammalian-adapted, avian-derived viruses (9–14). To our knowledge, there are no reports associating the other two mutations we identified, PA_K339M and PA_M561V in H5Aichi-P5, with mammalian adaptation. Idoko-Akoh et al. (15) produced gene-edited chickens with N129I and D130N substitutions in chANP32A and conducted an experimental infection with an H9N2 avian influenza virus. This resulted in breakthrough infections, with the collected virus possessing mouse-adaptation-related amino acid substitutions in PB2 and PA. Domingues et al. (16) suggested that viral passage in mammalian cells expressing ANP32A variants with partial or complete defects could drive selection for the PB2_E627K mutation in influenza A viruses. *In vivo* experiments by Liang et al. (17) demonstrated that H7N9 mutant viruses with the PB2_D701N mutation, a well-known marker for mammalian adaptation, were recovered from ANP32A-KO mice (18, 19). Consistent with these findings, our breakthrough Gs/Gd-lineage H5HPAI viruses replicating independently of chANP32A carried mammalian adaptation-related amino acid substitutions in the viral PB2 and PA genes. These results suggest that the acquisition of chANP32A-independent replication capacity is related to amino acid mutations associated with mammalian adaptation.

### ANP32A-independent replication of mutant Gs/Gd-lineage HPAI viruses may involve mechanisms other than increased polymerase activity

**Fig 4:**
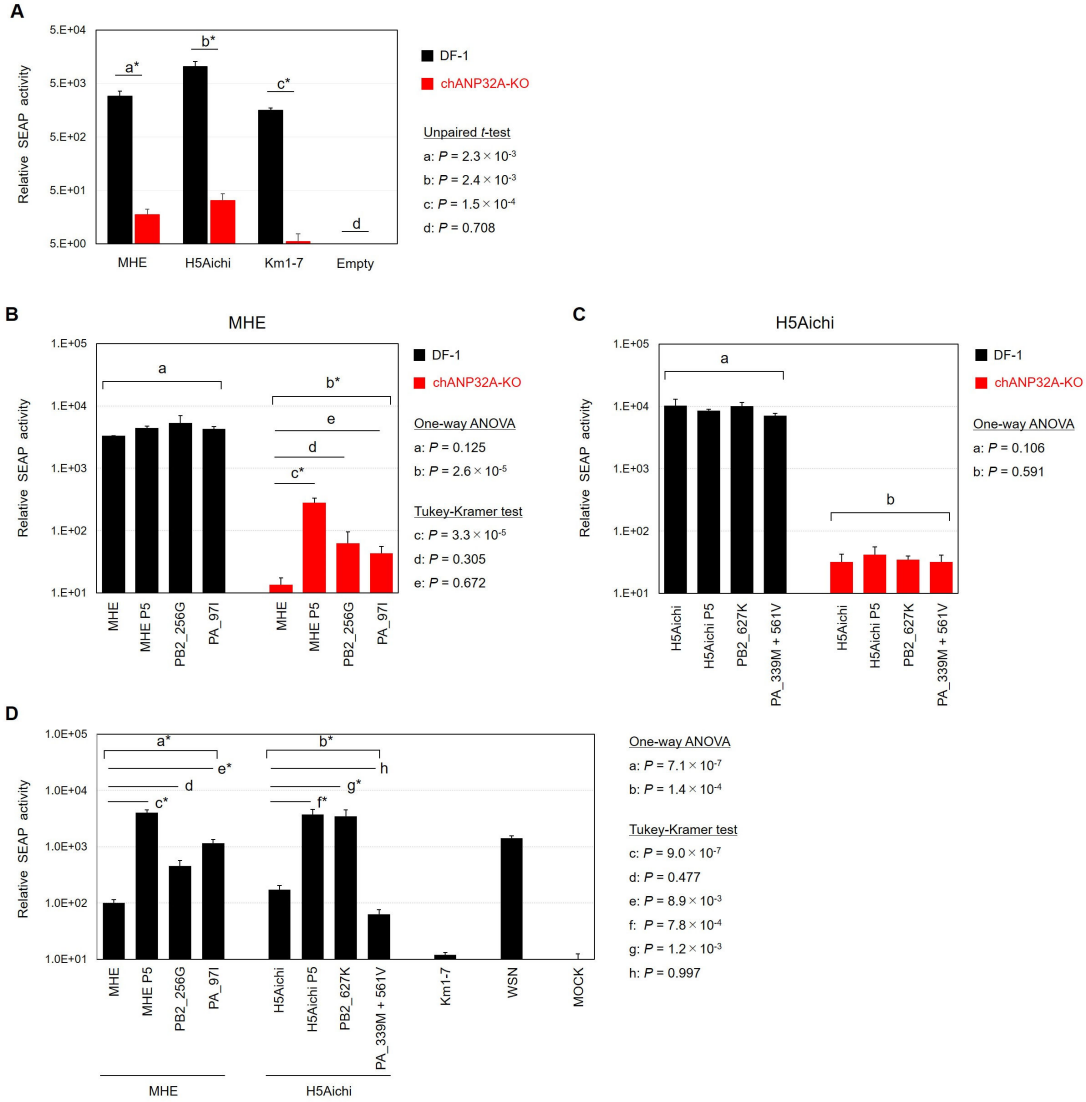
Effect of amino acid mutations on viral polymerase activity in HPAI virus mutants generated via serial passage in chANP32A-KO cells. (**A**) Comparison of the polymerase activities of the MHE, H5Aichi, and Km1-7 strains in wild-type DF-1 and chANP32A-KO cells. PB2, PB1, PA, and NP protein-expressing pCAGGS plasmids, the SEAP gene-expressing pPolIGG plasmid, and the secreted *Metridia* luciferase protein-expressing plasmid (pMetLuc2-vector) as a transfection control were co-transfected into cells seeded in 12-well plates. After 72 h, the cell supernatants were harvested, and SEAP and *Metridia* luciferase bioluminescence was measured. Differences in transfection efficiency between wells were corrected using the luciferase expression levels. The polymerase activity of each sample was determined from triplicate test results. Each bar is marked with the corresponding standard deviation. Statistical analysis of the differences in polymerase activity between wild-type DF-1 and chANP32A-KO cells was performed using an unpaired *t*-test. Asterisks indicate statistically significant differences (*P* < 0.05). (**B**) The effect of amino acid mutations found in the PB2 and PA proteins of MHE mutants on viral polymerase activity. The statistical significance of the differences in the polymerase activities between the parental MEH, MHE-P5, and their combinations in DF-1 or chANP32A-KO cells was determined via one-way ANOVA with the Tukey-Kramer test. Asterisks indicate statistically significant differences (*P* < 0.05). (**C**) The effect of amino acid mutations found in the PB2 and PA proteins of H5Aichi mutants on viral polymerase activity. (**D**) The effect of amino acid mutations found in the PB2 and PA proteins of MHE and H5Aichi strains on viral polymerase activity in HEK-293 cells.

The substitutions PB2_D256G, PB2_E627K, and PA_T97I have been reported to increase viral RNA polymerase activity in mammalian cells, enabling efficient replication of the viral genome and particles in infected mammalian hosts (9–11). These findings suggest a correlation between increased viral polymerase activity and enhanced replication efficiency of the influenza virus. To investigate whether mutant strains recovered polymerase activity in chANP32-KO cells, we assessed relative polymerase activities using reconstituted viral minigenomes. Initially, a reporter assay utilizing reconstituted minigenomes from the parental MHE, H5Aichi, and Km1-7 strains demonstrated that the polymerase activities of all strains were significantly diminished in chANP32A-KO cells compared to those in DF-1 cells (Fig. 4A). Subsequently, we evaluated the polymerase activities of reconstituted strains derived from the parental MHE strain, a passaged MHE strain (MHE-P5), and recombinant polymerase complexes with single mutations (MHE-PB2_D256G or MHE-PA_T97I) (Fig. 4B). In wild-type DF-1 cells, none of these reconstituted strains exhibited significant differences in polymerase activity. However, in chANP32A-KO cells, MHE-P5 exhibited significantly higher polymerase activity than the parental MHE strain. Neither polymerase complex with the single mutation demonstrated a significant increase in activity although a slight, non-significant increase was noted for each. These results suggest that the double mutations of PB2_D256G and PA_T97I are necessary for enhancing the polymerase activity of MHE-P5 in chANP32A-KO cells. However, in chANP32A-KO cells, the polymerase activity of MHE-P5 did not reach the same level as that observed in wild-type DF-1 cells.

Furthermore, we assessed the polymerase activity of minigenomes from the parental H5Aichi strain, a passaged H5Aichi strain (H5Aichi-P5), and recombinant polymerase complexes with single or double mutations (H5Aichi-PB2_E627K or H5Aichi-PA_K339M-plus-M561V) (Fig. 4C). The activities of these polymerase complexes in both wild-type DF-1 cells and chANP32A-KO cells showed no significant differences. These findings suggest that mechanisms other than increased polymerase activity dependent on chANP32A may contribute to the efficient viral replication of the H5Aichi-P5 strain in chANP32A-KO cells. Our findings are consistent with previous reports that a strict correlation between polymerase activity and virus replication is not always present (18, 20–22). Additionally, amino acid mutations in polymerase proteins not only influence polymerase activity but also facilitate evasion of the host’s natural immune response, thereby enhancing viral replicative capacity (23, 24). Given that the polymerase mutations identified in this study may contribute to chANP32A-independent replication, further analysis is needed to elucidate their underlying mechanism.

Subsequently, the polymerase activities of the MHE and H5Aichi strains were evaluated in HEK-293 cells, a human-derived cell line (Fig. 4D). Although the parental MHE and H5Aichi strains exhibited relatively high polymerase activities in HEK-293 cells compared to the Km1-7 strain, their activities were lower than that of the human-origin A/WSN/1933 (H1N1; WSN) strain. In contrast, passaged strains of MHE and H5Aichi (MHE-P5 and H5Aichi-P5, respectively) demonstrated significantly increased polymerase activity compared to their parental strains, reaching levels comparable to that of the WSN strain. For the MHE strain, neither the PB2-D256G nor the PA-T97I mutation alone achieved the polymerase activity level of MHE-P5, indicating that both mutations are essential for the enhanced polymerase activity of MHE-P5 in HEK-293 cells. For the H5Aichi strain, the PB2-E627K mutation alone resulted in polymerase activity comparable to that of H5Aichi-P5, while the PA double mutation (H5Aichi-PA_K339M-plus-M561V) did not show any enhancement in the activity, demonstrating that the enhanced polymerase activity of H5Aichi-P5 was primarily supported by the PB2-E627K mutation. These findings suggest that repeated infection of Gs/Gd-lineage H5HPAI viruses in chANP32A-KO cells may promote the introduction of amino acid mutations associated with adaptation to mammals.

In this study, we demonstrated that the deletion of chANP32A significantly reduces replication of Gs/Gd-lineage H5 HPAI viruses in chicken-derived cells. This finding highlights the potential of chANP32A as a host target for developing novel antiviral strategies. However, the emergence of mutant viruses capable of replicating independently of chANP32A raises a significant concern for the practical application of genetically engineered chickens. These mutant viruses acquired amino acid substitutions in the PB2 and PA genes, some of which are associated with mammalian adaptation. This suggests that, while chANP32A gene editing could be a promising approach for controlling avian influenza, there is a risk of generating viruses with enhanced replication capabilities in mammals. Although chANP32A is an attractive target gene, developing a strategy for influenza-resistant poultry may require the identification and integration of additional target genes. Further research is needed to fully understand the mechanisms underlying chANP32A-independent replication and to assess the long-term implications of using chANP32A gene-edited chickens for avian influenza control.

## MATERIALS AND METHODS

### Viruses and cells

The following five HPAI virus strains were used in this study: A/mountain hawk-eagle/Kumamoto/1/2007 (H5N1; MHE) (25), A/chicken/Aichi/2/2011 (H5N1; H5Aichi) (26), A/crane/Kagoshima/KU-4/2016 (H5N6; KU-4) (27), A/chicken/Kumamoto/1-7/2014 (H5N8; Km1-7) (28), and A/duck/KU-116/2015 (H5N8; KU-116) (29). The viral nucleotide sequences are available in the GenBank/GISAID database under accession numbers AB525188–AB525195 for MHE, AB684255 and AB684176–AB684182 for H5Aichi, EPI867577–EPI867584 for KU-4, AB932553–AB932560 for Km1-7, and EPI573677–EPI573684 for KU-116. These viruses were propagated in 10-day-old embryonated chicken eggs at 37°C for 2 days and then stored at −80°C prior to use. DF-1 cells purchased from the American Type Culture Collection (ATCC) were cultured in Dulbecco’s modified Eagle’s medium (DMEM; Gibco) containing 5% fetal bovine serum at 37°C in an atmosphere containing 5% CO_2_.

### Establishment of chANP32A-KO DF-1 cells

A chANP32A-KO DF-1 cell clone was generated by the use of CRISPR/Cas9. CRISPR target sequences were selected from exon 2 of chANP32A by use of CRISPRdirect (https://crispr.dbcls.jp). pSpCas9(BB)-2A-puromycin (pX459) was purchased from AddGene (Shanghai, China). The oligo DNAs C-ANP32A-2_F1 (5′-caccgacaactgtaggtcatacga-3′) and C-ANP32A-2_R1 (5′-aaacctgttgacatccagtatgct-3′) were annealed using a standard method. The resulting short double-stranded DNA was then inserted into the *Bbs*I restriction site of the pX459 vector using a DNA Ligation Kit (Takara Bio, Japan). DF-1 cells cultured in a 100 mm dish were transfected with 4 µg of pX459 containing a target sequence (5′-gacaactgtaggtcatacga-3′) complementary to exon 2 of chANP32A using METAFECTENE PRO (Biontex Laboratories, Munich, Germany). To select a single-cell chANP32A-KO DF-1 clone, transfected cells were cultured in DMEM containing 5% fetal bovine serum and 5 µg/mL of puromycin dihydrochloride (Sigma-Aldrich) with a cloning cylinder. To analyze the nucleotide sequence of the target gene, the genomic DNA of the generated DF-1 cell clone was extracted using NucleoSpin Tissue XS (MACHEREY-NAGEL GmbH&Co, KG, Germany). Complementary DNA containing the target genome sequence edited from exon 2 was amplified with TAKARA Ex Taq (Takara Bio, Japan), using the following primers: C-ANP32A-2_F2 (5′-tagcaaaattggggatcagg-3′) and C-ANP32A-2_R2 (5′-ctgccaacacttccagtcct-3′) and C-ANP32A-2_F3 (5′-ttggggatcaggcacagaaa-3′) and C-ANP32A-2_R3 (5′-cacttccagtcctcctgaga-3′). After purifying the PCR products using a Monarch Gel Extraction Kit (NEW ENGLAND BioLabs Inc.), the DNA sequences were analyzed using a BigDye Terminator v3.1 Cycle Sequencing Kit (Thermo Fisher Scientific) and the C-ANP32A-2_ F3 primer.

### Western blot analysis

Cells were lysed with RIPA buffer (ATTO), and the total protein was separated by the use of 10% SDS-PAGE and transferred onto PVDF membranes. The membranes were blocked with a mixture of 5% skim milk powder and PBS and then incubated with a primary antibody and an alkaline phosphatase-conjugated secondary antibody. Protein bands on the membrane were detected using Western Blue Stabilized Substrate for Alkaline Phosphatase (Promega). The antibodies in this study were purchased from commercial supplies. The primary antibodies included rabbit anti-ANP32A polyclonal antibody AB1 (Sigma-Aldrich) and mouse anti-beta actin monoclonal antibody AC-15 (Sigma-Aldrich). The alkaline phosphatase-conjugated secondary antibodies were goat anti-rabbit IgG antibody ab7091 (Abcam) and goat anti-mouse IgG antibody A3562 (Sigma-Aldrich).

### Determination of virus titers in cultured cells

Cells were seeded onto 48-well plates and inoculated into wells with 100 µL of a 10-fold serial dilution of viral sample per well. After incubation at 37°C for 1 h, the cells were washed twice with DMEM, and 200 µL of DMEM containing 0.5% bovine serum albumin (BSA) was added. At 3 days post-inoculation, the cells were observed for cytopathic effects (CPE), and viral titer was calculated using the Reed and Muench method. Viral titers are presented and expressed as the mean of three independent replicates.

### Passage of viruses in the chANP32A-KO cell

chANP32A-KO cells seeded onto 6-well plates were inoculated with a 10-fold serial dilution of a viral sample and incubated for 1 h at 37°C. The cells were then washed and maintained in DMEM supplemented with 0.5% BSA at 37°C. When CPE was apparent in the cells at 3 days post-inoculation, the CPE-positive culture supernatant was harvested from the well inoculated with the highest dilution of virus, and the harvested sample was used for the next passage in chANP32A-KO cells. After the collected virus was propagated once in embryonated chicken eggs, the virus titer of each passaged virus was determined by a TCID_50_ assay as described above.

### Minigenome assay

Viral polymerase activity was measured using a mini-genome reporter (pPolIGG/SEAP), which contains the secreted alkaline phosphatase (SEAP) gene flanked by non-coding regions of the AIV NP gene segment and is transcribed from pPolIGG plasmid encoding a chicken-specific polI promoter and a murine terminator sequence (30). We constructed pCAGGS expression plasmids encoding each polymerase component and NP for MHE, H5Aichi, and Km1-7. To measure viral polymerase activity, wild-type DF-1 or chANP32A-KO cells seeded in 12-well plates were transfected with pPolIGG/SEAP plasmid (250 ng), pCAGGS plasmids encoding the PB2 (250 ng), PB1 (250 ng), PA (250 ng), NP (250 ng) proteins, and 250 ng secreted *Metridia* luciferase expression plasmid (pMetLuc2-Control vector: Clontech Laboratories) as an internal control using a ScreenFect A plus transfection reagent (Wako) in accordance with the manufacturer’s instructions. Concurrently, the cells were also transfected with 250 ng empty pPolIGG, pCAGGS encoding PB2 (250 ng), PB1 (250 ng), PA (250 ng), and NP (250 ng) of MHE, and 250 ng pMetLuc2-Control vector. The cells were then incubated at 37°C. Forty-eight hours after transfection, the supernatant was harvested, and SEAP and *Metridia* luciferase bioluminescence were measured using a Ready-To-Glow Dual Secreted Reporter Vector Kit (Clontech Laboratories) with a GloMax-Multi Detection System (Promega).

Viral polymerase activity was measured in human-derived HEK293 cells in the same manner as described above. The reporter expression plasmid was a pHH21 plasmid containing the SEAP gene. The expression level of luciferase in each well of the assay did not show any statistically significant difference (data not shown).

### Statistical analysis

All statistical analyses were performed with EZR (Saitama Medical Center, Jichi Medical University, Saitama, Japan), which is a graphical user interface for R (The R Foundation for Statistical Computing, Vienna, Austria). More precisely, it is a modified version of R commander designed to add statistical functions frequently used in biostatistics.

## Data Availability

All data are available upon request.
